# Superparamagnetic Iron Oxide Nanoparticles Decorated Mesoporous Silica Nanosystem for Combined Antibiofilm Therapy

**DOI:** 10.3390/pharmaceutics14010163

**Published:** 2022-01-11

**Authors:** Elena Álvarez, Manuel Estévez, Alvaro Gallo-Cordova, Blanca González, Rafael R. Castillo, María del Puerto Morales, Montserrat Colilla, Isabel Izquierdo-Barba, María Vallet-Regí

**Affiliations:** 1 Departamento de Química en Ciencias Farmacéuticas, Faculdad de Farmacia, Universidad Complutense de Madrid, Instituto de Investigación Sanitaria Hospital 12 de Octubre i+12, 28040 Madrid, Spain; elealvar@ucm.es (E.Á.); manestev@ucm.es (M.E.); blancaortiz@ucm.es (B.G.); rafael.castillor@uah.es (R.R.C.); 2 CIBER de Bioingeniería Biomateriales y Nanomedicina CIBER-BBN, 28029 Madrid, Spain; 3 Instituto de Ciencia de Materiales de Madrid, ICMM/CSIC, Sor Juana Inés de la Cruz 3, 28049 Madrid, Spain; alvaro.gallo@csic.es (A.G.-C.); puerto@icmm.csic.es (M.d.P.M.)

**Keywords:** mesoporous silica nanoparticles, superparamagnetic iron oxide nanoparticles, thermo-responsive polymer coating, antibiotic delivery, combined therapy, bacterial biofilm

## Abstract

A crucial challenge to face in the treatment of biofilm-associated infection is the ability of bacteria to develop resistance to traditional antimicrobial therapies based on the administration of antibiotics alone. This study aims to apply magnetic hyperthermia together with controlled antibiotic delivery from a unique magnetic-responsive nanocarrier for a combination therapy against biofilm. The design of the nanosystem is based on antibiotic-loaded mesoporous silica nanoparticles (MSNs) externally functionalized with a thermo-responsive polymer capping layer, and decorated in the outermost surface with superparamagnetic iron oxide nanoparticles (SPIONs). The SPIONs are able to generate heat upon application of an alternating magnetic field (AMF), reaching the temperature needed to induce a change in the polymer conformation from linear to globular, therefore triggering pore uncapping and the antibiotic cargo release. The microbiological assays indicated that exposure of *E. coli* biofilms to 200 µg/mL of the nanosystem and the application of an AMF (202 kHz, 30 mT) decreased the number of viable bacteria by 4 log_10_ units compared with the control. The results of the present study show that combined hyperthermia and antibiotic treatment is a promising approach for the effective management of biofilm-associated infections.

## 1. Introduction

Bacterial infections pose a serious threat to public health, becoming the second leading cause of death worldwide [1,2,3], with biofilms being the main cause of most resistant infections [1,4]. Biofilms are communities of microorganisms that are covered by a protective extracellular matrix. This self-produced matrix protects the bacteria from hostile environmental conditions, reduces the efficacy of antibiotics compared with the effect in their planktonic counterparts and is responsible for the increased resistance to antimicrobials [4,5]. It has been shown that bacteria in biofilms can tolerate antibiotics at concentrations up to 1000 times higher than bacteria in a planktonic state [6,7]. Both the prevalence of antibiotic resistance and the increase in biofilm-associated infections are driving the demand for new, advanced and more effective treatments for such infections [8,9,10]. In this regard, nanotechnology offers an innovative platform to address this challenge [11]. Nanocarriers able to load, protect and locally deliver antimicrobial agents become ideal candidates for developing new nanomedicines [12,13,14]. Among them, mesoporous silica nanoparticles (MSNs) present attractive characteristics, such as their thermal/mechanical stability, adjustable pore size, high surface area, biocompatibility, low toxicity, ease of functionalization and capacity to host molecules inside their pores [15,16,17]. Furthermore, engineered and innovative recent formulations based in MSNs are envisioned as new nanoantibiotics for bacterial infection treatment [18]. For example, the combination of different antimicrobial elements within the same nanoplatform, such as the targeted co-delivery of antibiotics [19], or the combination of antibiotics plus multiactive metal ions [20] have been reported as advanced strategies in the fight against antimicrobial resistance. It is also feasible to functionalize MSNs with nanogates that can be opened to deliver antimicrobial cargo upon the application of an external or internal given stimuli, as has been widely reported [21,22,23]. External physical stimuli are gaining attention because they allow the control of antimicrobial release on demand. Thus, temperature [24,25], light [26] or alternating magnetic fields (AMF) [23] have been reported as promising release triggers.

Regarding the combination of different antimicrobial effects, it is well known that elevated temperatures, outside the optimal range of bacterial growth, inhibit bacterial proliferation and motility [27,28,29,30,31,32,33,34,35,36,37,38] by altering the structural integrity of biofilms [39,40], or reducing the rigidity of biofilms [40,41]. As a result, recent studies have focused on the use of hyperthermia as a physical method to make bacteria more sensitive to antimicrobial therapy [42,43,44,45,46,47,48]. Although to date, magnetic hyperthermia has been used mainly for the treatment of cancer [49,50,51,52], as a means to selectively ablate cancer cells without damaging the surrounding tissue [53,54], using hyperthermia to achieve an antibacterial effect is gaining increasing attention [55]. Superparamagnetic iron oxide nanoparticles (SPIONs) are one of the most effective heating materials for local hyperthermia when applying an AMF [56,57], as they absorb electromagnetic radiation when exposed to the high frequency field and convert it into localized heat [58,59]. The amount of heat generated by SPIONs not only depends on the size, shape, composition and aggregation state of the nanoparticles themselves, but also on the specific conditions (frequency and amplitude) of the applied AMF [60,61]. In this regard, different theoretical and experimental tests have revealed that, although the temperature generated on the surface of the SPIONs can reach up to the boiling point of the medium, it decays rapidly at a few nanometers from their surface [62,63,64]. Therefore, by tailoring such parameters it is feasible to optimize the local temperature induced in the surface of these SPIONs, affecting neither the surrounding media nor healthy tissues. Moreover, SPIONs in close contact with the biofilm would provoke the disruption of the polysaccharide matrix and biofilm removal through its rupture and detachment [65,66,67]. Due to their magnetic behavior, biocompatibility [68,69,70] and chemical stability, SPIONs are widely used in biomedical sciences, e.g., the management of Gram-negative bacterial infection, multimodal in vivo imaging, etc. [71,72,73,74]. However, the use of hyperthermia alone remains limited due to the potential thermal damage caused to the host tissue, as higher thermal doses are often required for the complete eradication of bacterial pathogens [75,76]. To help solve these problems, the use of hyperthermia as an adjuvant to antimicrobial treatment could facilitate the effective and rapid inactivation of the bacteria present in the biofilm, thereby reducing the duration of treatment and producing an improved effect by acting in a combined fashion [44].

Herein, we report on the design of a novel MSNs-based magnetic nanosystem to combine the AMF-triggered release of antibiotics and magnetic hyperthermia against bacterial biofilm. To this aim, MSNs were coated with a thermosensitive poly-*N*-isopropylacrylamide (PNIPAM) polymer with the ability to undergo a hydrophilic-to-hydrophobic (linear-to-globular) phase transition at a temperature between 40–43 °C. The outermost surface of the polymer-coated nanosystem was decorated with magnetite (Fe_3_O_4_) SPIONs, which serve as hot spots causing the shrinkage of the polymeric network. When the nanosystem is loaded with levofloxacin, this effect triggers the release of the antibiotic cargo [62]. The clever design of the nanosystem, with SPIONs decorating the outermost surface, may allow the close contact of the magnetic elements with the target, i.e., the biofilm, improving the bactericidal effect because the heating is directly on the biofilm zone and is not shielded by the silica structure. In addition, the slight antimicrobial activity of the SPIONs themselves [77] may be enhanced by the developed nanosystem. This proof-of-concept based on the combination of magnetic-hyperthermia therapy and thermo-responsive antibiotic delivery throughout the smart assembly of different functional building blocks into a mesoporous silica-based nanoplatform paves the way towards the design of new nanomedicines for local antibiofilm therapies.

## 2. Materials and Methods

### 2.1. Reagents and Equipment

Tetraethylorthosilicate (TEOS), cetyltrimethylammonium bromide (CTAB), levofloxacin (LEVO, L), sodium oleate 82%, FeCl_3_·6H_2_O 97%, oleic acid 90%, octadecene 90%, ammonium nitrate, 3-aminopropyltriethoxysilane 97% (APTES), 3-(trimethoxysilyl)propyl methacrylate (MPS), phosphate-buffered saline (PBS), *N*-isopropylacrylamide (NIPAM), *N*,*N’*-methylenebis(acrylamide) (MBA), ammonium persulfate (APS), citric acid monohydrate ≥99.0%, *N*,*N*-diisopropylethylamine 99.5%, *N*-hydroxymethyl-acrylamide (NHMA, 48% wt. solution in water), piperidine ≥99.5%, fluorenylmethoxycarbonyl chloride (Fmoc-Cl), α-(9-fluorenylmethyloxycarbonyl)amino-27(ethyleneglycol)-omega-propionic acid (Fmoc-NH-(PEG)_27_-COOH) and Luria–Bertani broth (LB) were purchased from Sigma Aldrich (Madrid, Spain). All other chemicals (sodium carbonate monohydrate, *N*,*N*-dimethylformamide (DMF), absolute ethanol, acetone, hexane, etc.) were of the highest quality commercially available and used as received. Deionized water was further purified by passage through a Mili-Q Advantage A-10 purification system (Milipore Corporation, Burlington, MA, USA) to a final resistivity of 18.2 MΩ cm.

The analytical methods used to characterize the synthesized compounds were as follows: powder X-ray diffraction (XRD), thermogravimetry (TGA), Fourier transformed infrared (FTIR) and fluorescence spectroscopies, transmission electron microscopy (TEM), energy dispersive X-ray spectroscopy (EDS), electrophoretic mobility measurements to calculate the values of zeta-potential (ζ), dynamic light scattering (DLS), vibrating sample magnetometry (VSM) and hyperthermia measurements. The equipment and conditions used are described in the Supplementary Materials.

### 2.2. Synthesis of Materials

#### 2.2.1. Synthesis of Magnetic Nanoparticles (Fe_3_O_4_ NPs)

Magnetite nanoparticles (Fe_3_O_4_ NPs) were synthesized through a two-step procedure (Figure S1). The first synthetic step consisted of the thermal decomposition of an iron oleate complex in octadecene [78]. To prepare the iron precursor, 45 g of sodium oleate and 10.8 g of FeCl_3_·6H_2_O were added into a 500 mL single-mouth round bottom flask and mixed with 60 mL of distilled water, 80 mL of absolute ethanol and 140 mL of cyclohexane. The mixture was stirred for 4 h at 70 °C under reflux. After this time, the reaction was allowed to cool down to room temperature (RT) and the two distinguishable phases were separated by using a funnel. The organic phase containing the iron oleate was washed with an ethanol:water 50:50 (*v*/*v*) mixture (50 mL × 3 times) and the hexane was evaporated using a rotary evaporator, also removing ethanol and water residues from the washings. Hereinafter, the Fe_3_O_4_ NPs were prepared, using 4.5 g of the as prepared iron oleate and 0.71 g of oleic acid mixed with 50 mL of octadecene in a three-neck round bottom flask. A nitrogen flow and a refrigerant were connected to the flask and the mixture was stirred and heated up to 315 °C. At 50 °C, the stirring was stopped and at 100 °C the nitrogen flow was shut off. Once 315 °C was reached, the reaction was left for 1 h. Finally, the mixture was cooled and the sample was washed 4 times with ethanol by centrifugation (10,000 rpm during 5 min), and redispersed in toluene. In the second synthetic step, the nanoparticles suspended in the organic solvent were transferred to water via a ligand exchange method [79]. This process was performed using citric acid (0.05 g) in a DMF:chloroform solution 50:50 (*v*/*v*) (15 mL). Briefly, 15 mg of the synthesized NPs were placed in contact with the citric acid solution and kept overnight at 30 °C under stirring. The obtained product was then washed once with DMF to remove excess citric acid and then three times with ethanol, and left to dry at RT. In this way, we obtained the magnetic nanoparticles denoted as Fe_3_O_4_ NPs (Figure S1).

#### 2.2.2. Synthesis of Mesoporous Silica Nanoparticles (MSNs)

The synthesis of mesoporous silica nanoparticles was carried out following the modified Stöber method [80]. For this purpose, 1 g of CTAB (2.74 mmol) was dissolved in 480 mL of water and 3.75 mL of NaOH 2 M and heated to 80 °C. Then, a solution containing 5 mL of TEOS (22.4 mmol) was added to the previous solution under vigorous stirring using a syringe dispenser at a constant rate of 0.26 mL/min. The stirring and temperature were maintained for 2 h, and then the suspension was cooled to RT. The solid particles were isolated and washed by centrifugation (10,000 rpm during 5 min) several times with water and EtOH and finally dried. The nanoparticles still containing the surfactant (CTAB) inside the mesopores were denoted as MSN*. The surfactant-free MSNs were obtained by removing the organic template through an ion exchange method by suspending 180 mg of MSN* into 60 mL of an extracting solution composed of NH_4_NO_3_ (10 mg/mL), EtOH (95%, *v*/*v*) and water (5%). The mixture was heated to 75 °C and stirred overnight. Then, the nanoparticles were washed with water (once) and ethanol (three times). The extraction process was repeated two more times and the obtained solid was dried.

#### 2.2.3. Grafting of Polyethyleneglycol to MSNs (MSN_f_-PEG)

The external surface of MSNs* was functionalized with amino and methacrylate groups using APTES and MPS, respectively, as reagents. For this purpose, 0.1 g of MSN* was dispersed in a solution of APTES (33.3 µL) and MPS (50 µL) (relation 2:3, *v*/*v*) in absolute ethanol (15 mL). The mixture was left under magnetic stirring at 45 °C for 24 h, and the nanoparticles were recovered by centrifugation and washed with ethanol. The solid (MSN_f_*) was dried at 37 °C for 24 h. Later, the surfactant was removed from the interior of the pores following the procedure described in Section 2.2.2 to obtain MSN_f_. Subsequently, the PEG-derivative, Fmoc-NH-(PEG)_27_-COOH (M.W. 1544.8 g/mol), was grafted to the surface of MSN_f_ via reaction with the amino groups in the external surface of surfactant-free functionalized nanoparticles (MSN_f_). The number of accessible amino groups on MSN_f_ was quantified by using Fmoc-Cl as described elsewhere [81] to calculate the amount of Fmoc-NH-(PEG)_27_-COOH to be added. Thus, 2 mL of a suspension of MSN_f_ in DMF (4 mg/mL) was reacted with 68.8 mg Fmoc-Cl (overnight, RT and magnetic stirring). Subsequently, the excess of unreacted Fmoc-Cl was removed with DMF (4 × 10 min). Later, the amino groups were deprotected (NH-Fmoc). For this, the nanoparticles are placed in contact with a solution of DMF (20% piperidine) for 3 h under magnetic stirring in such a way that the starting nanoparticles with free amino groups were recovered by centrifugation, whereas 9-methylene-9H-fluorene (Fmoc) remained in the supernatant. The absorbance of the latter measured at 301 nm was then interpolated in a previously recorded calibration curve to determine the amount of Fmoc-Cl. Since there was an equimolar Fmoc-Cl:NH_2_ ratio, the number of amino groups on the surface of MSN_f_ was also known, which allowed the determination of the amount of PEG derivative to be added. Briefly, a mixture of 1 eq of MSN_f_, 1.2 eq Fmoc-NH-(PEG)_27_-COOH, 1.5 eq EDC and 3 eq DIPEA in DMF as solvent was kept at RT overnight under magnetic stirring. Then, the nanoparticles were washed with DMF (twice) and ethanol (twice) and left to dry in an oven at 37 °C. In this way, we obtained the MSN_f_-PEG sample.

#### 2.2.4. Polymerization of *N*-Isopropylacrylamide (MSN_f_-PEG-PNIPAM)

The radical polymerization was carried out using a 90:10 NIPAM to NHMA monomers ratio to obtain a polymer which undergoes a polymer transition from linear to globular in the 41–43 °C temperature range, as reported by Guisasola et al. [62]. Briefly, in a 100 mL three-neck round bottom flask, NIPAM (150.9 mg, 1.33 mmol), MBA (12 mg, 0.078 mmol), NHMA (49.4 µL, 0.148 mmol), CTAB (3.6 mg) and Na_2_CO_3_ (5 mg) were added to 45 mL of water. The solution was stirred under nitrogen bubbling at 70 °C for 30 min to remove all oxygen. Then, 50 mg of MSN_f_-PEG redispersed in 5 mL of absolute EtOH was added under the N_2_ stream and the solution was maintained for another 15 min. To initiate the polymerization of the monomer, 0.2 mL of a 35 mg/mL APS solution in previously deoxygenated water was added to the reaction mixture. Subsequently, the reaction mixture was allowed to cool and stirred overnight at RT. The mixture was centrifuged and washed 3 times with water to remove any unreacted monomers and finally allowed to dry, obtaining MSN_f_-PEG-PNIPAM^♦^. Afterwards, the Fmoc from the PEG derivative grafted to the nanoparticles was deprotected. For this purpose, MSN_f_-PEG-PNIPAM^♦^ sample (50 mg) was put in contact with 20 mL of a piperidine solution in DMF (20% vol.) for 3 h. Subsequently, the nanoparticles were washed to remove residual piperidine with DMF (×1) and ethanol (×3). In this way, the resulting nanosystem (MSN_f_-PEG-PNIPAM) exhibited free amino groups available for the subsequent incorporation of the Fe_3_O_4_ NPs.

#### 2.2.5. Grafting of Fe_3_O_4_ NPs to MSN_f_-PEG-PNIPAM (mMSN_f_-PEG-PNIPAM)

Of the MSN_f_-PEG-PNIPAM nanosystem, 30 mg was added to 15 mg of the Fe_3_O_4_ nanocrystals dispersed in ethanol and the mixture was kept overnight at 30 °C under stirring. Subsequently, the nanoparticles were retrieved by centrifugation and washed with ethanol (×3) to remove free Fe_3_O_4_ NPs, obtaining a mMSN_f_-PEG-PNIPAM sample.

### 2.3. Levofloxacin Loading and Triggered Release

#### 2.3.1. Loading of Levofloxacin (mMSN_f_-PEG-PNIPAM-L)

Of the mMSN_f_-PEG-PNIPAM material, 15 mg was soaked in 5 mL of a 0.008 M solution of levofloxacin (LEVO) in EtOH and the suspension was shaken under orbital stirring for 16 h in dark at 50 °C to bring the polymer into globular conformation and allow the antibiotic to enter the pores. The sample was then cooled to favor the polymer adopting the expanded conformation to close the pore entrances and prevent cargo release, then centrifuged, washed with EtOH and dried to obtain the mMSNf-PEG-PNIPAM-L sample.

#### 2.3.2. In Vial Triggered Levofloxacin Release Assays

Temperature as release trigger: The kinetic studies of LEVO release were performed in PBS 1× at different temperatures (20, 37 and 50 °C) and physiological pH 7.4. A double-chamber cuvette with two distinct compartments for sample and analysis was used for the experiments. The compartments were separated by a dialysis membrane (0.4 µm) that only allowed the diffusion of LEVO molecules. For this purpose, 0.68 mL of a suspension of the mMSN_f_-PEG-PNIPAM-L material in PBS (2.5 mg/mL) was placed in the sample compartment and 3.1 mL of fresh PBS in the analysis compartment. The volume of PBS placed in the analysis compartment was renewed at each measurement time. The amount of antibiotic released was measured by fluorescence spectroscopy using a Biotek Powerwave XS spectrofluorimeter (Gen5 software version 1.00.14). For the analysis of the released LEVO, λ_ex_ = 292 nm and λ_em_ = 494 nm were used and the calibration curve was recorded in a concentration range of 0.002 to 20 mg/mL. The experiment was carried out on three different release plates in independent incubators for each of the assayed temperatures 50, 37 and 20 °C.

Magnetic field as release trigger: A second release experiment was performed by exposing the mMSN_f_-PEG-PNIPAM-L nanosystem to an AMF (202 kHz, 30 mT). For this purpose, 0.5 mL of a suspension of LEVO-loaded nanosystem in PBS (1.96 mg/mL) and 4 mL of PBS were added to a well. The application of the magnetic field was performed at 30 min intervals, until reaching a final time of 240 min. After each 30 min time interval, the amount of LEVO released from the nanosystem at 37 °C without AMF and with AMF application was measured.

### 2.4. Microbiological Assays

#### 2.4.1. Bacterial Culture

*Escherichia coli* (Laboratory strain *E. coli* ATCC 25922) was used as a Gram-negative bacterial model for the assays. The *E. coli* bacteria were cultured by inoculation in Luria–Bertani (LB) broth and incubated for 24 h at 37 °C with orbital shaking at 100 rpm. After this time, 10 µL of the previous solution was inoculated in fresh LB medium and left to grow for 2 h. After that time, the bacteria were centrifuged for 10 min at 3500× *g* at 22 °C. The supernatant was discarded and the pellet was washed with PBS 3 times. The bacteria were then suspended and diluted in PBS to obtain a concentration of 2 × 10^9^ bacteria/mL and then diluted with the corresponding broth to obtain 2 × 10^6^ bacteria/mL. The concentration of bacteria was determined using a visible spectrophotometer (Photoanalizer D-105, Dinko instruments, Barcelona, Spain).

#### 2.4.2. Biofilm Growth

*E. coli* biofilms were grown prior to assays on 55 × 14 mm-size Petri dishes and by adding 2 mL of a bacterial suspension of 10^6^ bacteria per mL. The plate was maintained for 48 h at 37 °C, adding 0.5 mL of fresh medium after the first 24 h. After 48 h, each plate was thoroughly washed twice with 2 mL of PBS 1× buffer solution under sterile conditions to remove medium and non-biofilm-forming bacteria. The antimicrobial activity of the mMSN_f_-PEG-PNIPAM-L nanosystem against *E. coli* bacterial biofilms was quantitatively evaluated by determining the reduction in log_10_ (CFU/mL) (CFU = colony-forming units) to assess the viability of the biofilm.

#### 2.4.3. Biofilm Viability Assay

*E. coli* biofilms were preformed during 48 h as described above and then 1 mL of a mMSN_f_-PEG-PNIPAM-L suspension in LB at a concentration of 200 µg/mL was added. For comparative purposes, the performance of the LEVO-free nanosystem (mMSN_f_-PEG-PNIPAM) was also tested. A neodymium magnet was placed under the test Petri dish for 30 min prior to the application of the AMF. Exposure of the preformed *E. coli* biofilms to LEVO-containing and LEVO-free nanosystems was performed for one hour at 37 °C with application of the AMF. After incubation, the medium was removed from the Petri dishes, which were washed once with 2 mL of sterile PBS 1× and another 2 mL of fresh PBS 1×. Subsequently, mechanical disruption of the biofilm was performed with a sterile wooden tongue depressor and sonication was applied for 5 min in a low-power ultrasonic bath to break up and disperse the biofilm in a total volume of 2 mL of PBS 1×. The presence or not of bacteria, as well as their quantification, was determined by counting CFUs per mL using the droplet plate method on LB agar plates. The dilutions used were 1:100, 1:1000 and 1:10,000 in PBS 1× of the bacteria exposed to the nanosystems were made and five drops (5 × 10 µL) of each solution were inoculated in LB agar plates divided into three sectors, which were incubated for 16 h at 37 °C. The procedure described in reference [82] was followed for the mean count of the five drops of each dilution and the average counting for all dilutions was calculated. Log_10_(CFUs/mL) compared with bacteria without treatment after 16 h as control was determined. All assays were performed in triplicate with respective controls.

## 3. Results and Discussion

### 3.1. Synthesis and Physicochemical Characterization of the Nanosystem

**Figure 1:**
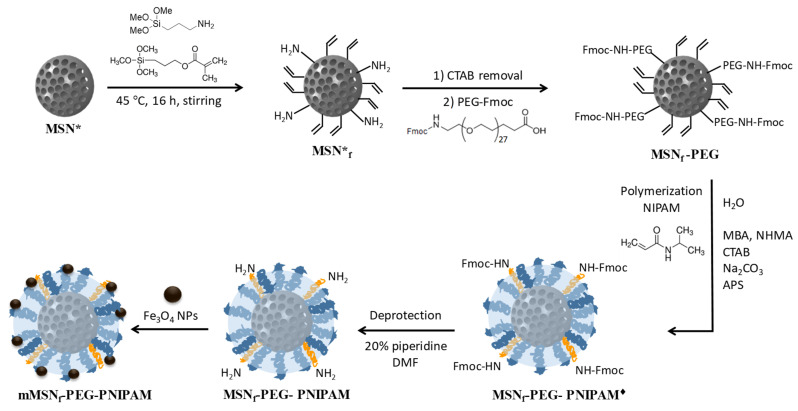
Synthetic steps carried out to obtain the mMSN_f_-PEG-PNIPAM nanosystem: MSNs with the surfactant inside the mesopores were used as starting materials (MSN*); surface modification of MSN* with methacrylate and aminopropyl groups by using MPS and APTES, respectively (MSN_f_*); surfactant removal followed by grafting of Fmoc-NH-(PEG)_27_-COOH (MSN_f_-PEG); polymerization of *N*-isopropylacrylamide (MSN_f_-PEG-PNIPAM^♦^); deprotection of Fmoc to leave amino groups available (MSN_f_-PEG-PNIPAM); and grafting of Fe_3_O_4_ NPs to MSN_f_-PEG-PNIPAM (mMSN_f_-PEG-PNIPAM).

The hybrid nanosystem designed in this research work was prepared through different synthetic steps, independently obtaining the polymer-functionalized MSNs and the SPIONs, to proceed with the incorporation of magnetic nanoparticles on the surface of the nanosystem at the end (Figure 1).

**Figure 2:**
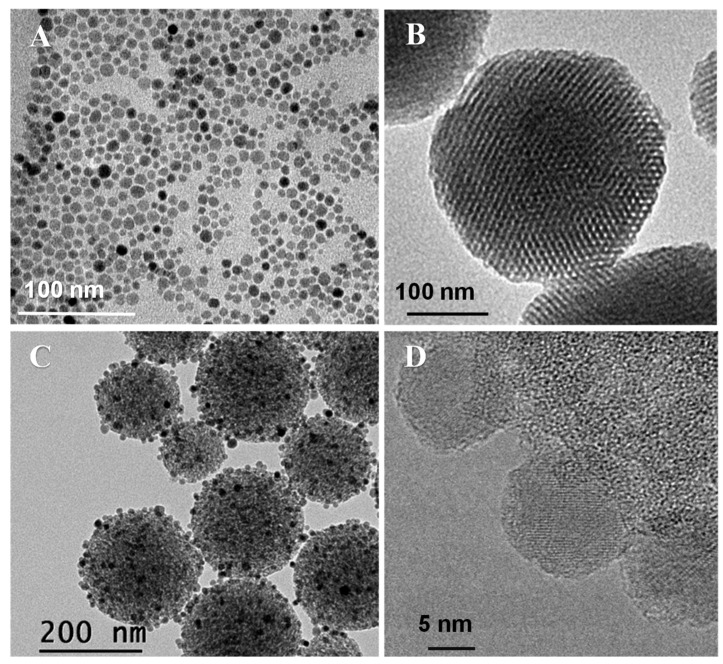
TEM images of OA-Fe_3_O_4_ NPs (**A**), bare MSNs (**B**), mMSN_f_-PEG-PNIPAM nanosystem (**C**) and HRTEM image of mMSN_f_-PEG-PNIPAM (**D**).

The synthetic procedure to obtain Fe_3_O_4_ NPs involved a two-step process consisting of a thermal decomposition of the Fe(oleate)_3_ precursor followed by ligand exchange using citric acid (Figure S1) [78,79]. The iron oleate complex was first prepared by reacting FeCl_3_.6H_2_O and sodium oleate. Freshly prepared Fe(oleate)_3_ was mixed with oleic acid and 1-octadecene and the mixture was submitted to thermal decomposition, to obtain oleic acid-stabilized F_3_O_4_ NPs (OA-Fe_3_O_4_ NPs) as a colloidally stable suspension in toluene. OA-Fe_3_O_4_ NPs were analyzed by TEM, revealing the presence of nanocrystals with a relatively uniform spherical morphology (Figure 2A). The statistical treatment of NP sizes from several TEM images revealed a narrow size distribution of particle diameter centered at 12 ± 2 nm, assuming a log-normal distribution to make the fit in the histogram (Figure S2A). The XRD pattern of these NPs pointed to a pure crystalline magnetite (Fe_3_O_4_) structure (Figure S2B), and by using the Scherrer´s equation [83], the crystal size was estimated (10 nm). To evaluate the magnetic behavior of these OA-Fe_3_O_4_ NPs, they were characterized under a direct current (DC) magnetic field. The DC experiments showed loops with coercive fields close to zero and saturation magnetization values of 97.4 emu/g_Fe_ close to the bulk value for magnetite (~100 emu/g_Fe_), demonstrating the superparamagnetic-like behavior of the synthetized OA-Fe_3_O_4_ NPs at RT (Figure S2C) [84].

The hydrophobic character of the synthesized OA-Fe_3_O_4_ NPs required a transfer step to aqueous medium for their subsequent anchoring to the mesoporous silica nanoparticles (Figure S1) [79]. For this purpose, the hydrophobic ligand (oleic acid) present in the OA-Fe_3_O_4_ NPs was exchanged with hydrophilic ligands bearing carboxylic groups that present affinity towards the magnetite surface. Herein, citric acid was used as the phase transfer agent, which may be adsorbed on the surface of magnetite nanoparticles through coordinating via one or two of the carboxylate groups depending on steric hindrance and also on the curvature of the surface [85]. This preserves at least one carboxylic acid group exposed to the aqueous medium, providing a surface of magnetite of hydrophilic character and giving rise to stable suspensions in water. The resulting samples were denoted as Fe_3_O_4_ NPs.

The second part of the synthesis of the hybrid nanosystem deals with the assembly of the different building blocks in the mesoporous silica nanoplatform (Figure 1). Firstly, the mesoporous silica nanoparticles still containing the surfactant inside the mesopores (MSN*) were employed as starting material for the subsequent grafting of APTES and MPS to promote the preferential functionalization of the external surface of nanoparticles.

On the one hand, APTES provided the nanosystem of –NH_2_ groups onto the surface of the MSNs for the successive PEGylation of MSN_f_ by condensation reaction with the –COOH groups from the polyethyleneglycol derivative (Fmoc-NH-(PEG)_27_-COOH) via carbodiimide chemistry [86]. The grafting of PEG into the nanoparticles provides several advantages, such as maintaining the nanoparticles’ dispersibility, improving their long-term colloidal stability in different biological media and increasing their circulation times [87]. Of foremost significance is the fact that PEG serves as a flexible extender spacer arm for the subsequent anchorage of the Fe_3_O_4_ NPs, so they preferentially locate in the outermost periphery of the nanosystem to be more accessible for direct contact with the targeted biofilm. In addition, the chosen PEG derivative contains a Fmoc-protected amine terminal group, which also serves as an anchoring point for the Fe_3_O_4_ NPs grafting after deprotection.

On the other hand, the polymerizable methacrylate groups of MPS present on the surface of MSN_f_-PEG allowed the growth of the thermosensitive polymer (PNIPAM) onto the outer surface of the nanosystem. The radical polymerization process was carried out by using different reactants: MBA, as the crosslinking agent to create a more branched polymeric network that hinders the premature cargo release from the mesopores; a monomers mixture in a 90:10 NIPAM to NHMA molar ratio; and APS as initiator. The amount of attached polymer was 4.9% as determined by TGA. Thermosensitive polymers based on PNIPAM are widely used for drug delivery applications because they are able to undergo a transition from linear (swelling state) to globular (collapsed) conformation when heated above their lower critical solution temperature (LCST) and they exhibit good biocompatibility. The use of NHMA, as a hydrophilic co-monomer, allows the polymer transition temperature to be increased up to 42–45 °C, as it increases the polymer–water interactions necessary for the conformational change [62].

Finally, once the PNIPAM-based polymer was grown into the mesoporous silica nanosystem, the PEG-Fmoc was deprotected with a piperidine solution which allowed the amino groups to be free for the subsequent incorporation of the iron oxide nanoparticles, endowed with carboxylic groups. To reach this goal, the Fe_3_O_4_ NPs were incubated overnight at 37 °C in ethanol with the MSN_f_-PEG-PNIPAM nanosystem, to obtain mMSN_f_-PEG-PNIPAM. The immobilization of Fe_3_O_4_ NPs on the surface of MSN_f_-PEG-PNIPAM can be probably ascribed to the combination of two main types of interactions. On the one hand, to the possible formation of hydrogen bonds taking place between the unbounded carboxylic acid groups (-COOH) of citric acid moieties onto the surface of Fe_3_O_4_ NPs and the amine and/or amide groups in the polymeric shell of the nanosystem. On the other hand, to hydrophobic interactions between the residual alkyl chains of the oleic acid units remaining on the surface of Fe_3_O_4_ NPs and the hydrocarbon chains of the PNIPAM polymer onto the mesoporous nanoparticles (see FTIR discussion below).

The bare MSNs are uniform in size with a mean diameter of 160 nm, confirmed by DLS (Table 1). The well-ordered mesoporous structure of MSNs is clearly observed in the TEM images (Figure 2B). After the successive functionalization steps, subsequent polymer growth and immobilization of the 12 nm Fe_3_O_4_ NPs, hardly any alteration of the morphology was observed. In addition, it is observed that the hydrodynamic nanosystem size increases up to 255 nm compared with the bare MSN (Table 1). Moreover, no fusion of nanoparticles is observed in the TEM images (Figure 2C). The amount of iron incorporated into the mMSN_f_-PEG-PNIPAM nanosystem was estimated by EDS (Figure S3). The atomic percentages of Fe and Si obtained were 7.2 ± 0.6 and 92.8 ± 0.4, respectively, resulting in a Fe/Si molar ratio of 0.0776.

**Figure 3:**
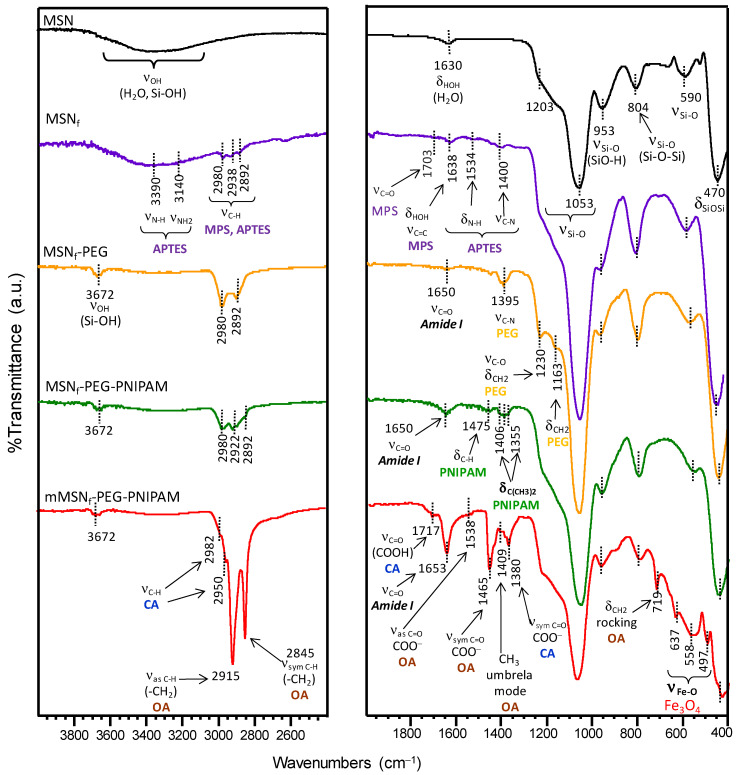
FTIR characterization of the different functionalization stages performed to obtain the final nanosystem mMSN_f_-PEG-PNIPAM.

A deep characterization of the different nanoparticles derived from the successive functionalization steps conducted in this research work (see Figure 1) was carried out by FTIR spectroscopy (Figure 3) and ζ-potential measurements (Table 1). As reference, the surfactant-free MSN material was added to the study. Figure 3 shows FTIR spectra of the different samples. As can be observed, all spectra display a broad band in the 3000–3600 cm^−1^ region, which can be attributed to the overlapping of the O-H stretching bands of hydrogen-bonded water molecules (H-O-H) and surface silanol groups (SiO-H) [88]. Si-O in-plane stretching vibrations of the silanol Si-OH groups appear at 953 cm^−1^. The intense Si-O covalent bond vibrations appear predominantly in the 1200–1000 cm^−1^ range, evidencing the existence of a dense silica network, where oxygen atoms serve as bridges between two silicon sites. Likewise, the symmetric stretching vibrations of Si-O-Si appear at ca. 800 cm^−1^ and its bending mode is observed at 470 cm^−1^. Finally, the low-intensity band at 590 cm^−1^ is assigned to Si-O stretching of the silica network defects [88].

The spectrum of the surfactant-free APTES and MPS functionalized material (MSN_f_) displays several bands in the 2892–2980 cm^−1^ range, assigned to C-H asymmetric and symmetric stretching vibrations of the alkyl chains of the organosilane precursors. Additional vibration bands at 3390 cm^−1^ (NH_2_ stretching), 3140 cm^−1^ (N-H stretching), 1534 cm^−1^ (NH deformation) and 1400 cm^−1^ (C-N stretching) confirm the presence of aminopropyl groups from APTES. On the other hand, the bands at 1703 cm^−1^, (C=O stretching) and 1638 cm^−1^ (C=C stretching) account for the successful incorporation of MPS [89].

ζ-potential measurements in water support this finding, since there is a severe change from negative (−22 mV) in bare MSN to +47 mV in MSN_f_ (Table 1). These values are due to the deprotonation of silanol groups in the MSN surface (Equation (1)) and protonation of primary amine groups in APTES after functionalization (Equation (2)), taking into account that the methacrylate groups do not virtually modify the surface charge [90,91].

R-Si-OH + H_2_O ⇌ R-Si-O^−^ + H_3_O^+^    pK_a_ ≈ 6.8 (1)

–NH_2_ + H_2_O ⇌ –NH_3_^+^ + OH^−^         pK_a_ ≈ 10.4 (2)

The success of the PEGylation procedure by the condensation reaction of –NH_2_ groups on the surface of MSN_f_ and carboxylic acid groups from the PEG derivative (Fmoc-NH-(PEG)_27_-COOH) via carbodiimide chemistry was also confirmed by FTIR and ζ-potential measurements. Thus, the FTIR spectrum of MSN_f_-PEG (Figure 3) displays new bands at 1650 cm^−1^ (C=O asymmetric stretching of amide group), 1163 cm^−1^ (C–O stretching and CH_2_ rocking) and 1230 cm^−1^ (CH_2_ twisting) [92].

The appearance of such new bands is accompanied by a noticeable increase in the intensity of vibration bands at ca. 1400 cm^−1^ (C-N stretching) and those in the 2892–2980 cm^−1^ range (C-H stretching) compared with those present in MSN_f_. Moreover, the partial consumption of the surface –NH_2_ groups present in MSN_f_ by reaction with the –COOH groups of the PEG-derivative explains the slight decrease in the obtained ζ-potential value to +34 mV for MSN_f_-PEG (Table 1).

The successful growth of the PNIPAM derivative by the radical polymerization of the above-mentioned monomers with the methacrylate groups from MPS onto the surface of MSN_f_-PEG was confirmed by FTIR. Figure 3 shows an increase in the intensity of the characteristic asymmetric stretching amide band at 1650 cm^−1^ and the appearance of new bands at 1475 cm^−1^ (C-H bending) and the doublet at 1406 and 1355 cm^−1^ attributed to the deformation of the isopropyl group (C(CH_3_)_2_) [62,93].

The ζ-potential value for this nanosystem would be expected to increase due to the presence of terminal –NH_2_ groups of PEG chains after the deprotection process. Nonetheless, the ζ-potential value for this sample decreases to 9 mV after the radical polymerization process, which can probably be ascribed to the partial masking of –NH_2_ groups by the globular PNIPAM polymeric chains grown onto the nanosystem surface.

In the final step of the process, Fe_3_O_4_ NPs were incorporated into MSN_f_-PEG-PNIPAM to obtain the final nanosystem, denoted as mMSN_f_-PEG-PNIPAM. In the FTIR spectrum of this nanosystem, the appearance of well-defined characteristic vibration bands confirms the successful incorporation of the magnetic nanoparticles (Figure 3). The different vibration bands appearing at 637 and 558 cm^−1^ can be attributed to the lattice stretching vibration modes of Fe-O bonds in tetrahedral and octahedral sites, whereas the band at 497 cm^−1^ could correspond to the vibration band of Fe-O of bulk magnetite [94]. Moreover, the FTIR spectrum of mMSN_f_-PEG-PNIPAM shows different vibration bands that are assigned to both citric acid (CA) and oleic acid (OA), which accounts for a partial ligand exchange of OA moieties by CA in Fe_3_O_4_ NPs (see Figure S1, Supporting Information). Among the different bands, noteworthy are those appearing at 1538 and 1465 cm^−1^, corresponding, respectively, to the asymmetric and symmetric C=O stretching of –COO^−^ groups, which revealed that OA was chemically adsorbed onto the surface of Fe_3_O_4_ nanoparticles through chemical interaction between their –COO^−^ groups and Fe atoms [95]. In addition, the band at 1717 cm^−1^, ascribed to the C=O stretching of –COOH, and the band at 1380 cm^−1^ assigned to the symmetric C=O stretching of –COO^−^ groups of CA, points to the bond of a unique carboxylate group to iron, as previously reported [88,96]. The presence of unbounded –COOH groups in the Fe_3_O_4_ NPs able to interact with amide groups by hydrogen bond interactions would account for their immobilization into the PNIPAM and PEG polymeric branches. This fact is supported by FTIR, where the significant increase in the intensity of the vibration band (C=O stretching) of the amide group at 1653 cm^−1^ points to the existence of O_carboxyl_H···O_amide_ hydrogen bonds [97].

The negative ζ-potential value of −6 mV measured for the mMSN_f_-PEG-PNIPAM is also in good agreement with the presence of –COOH in the surface of the immobilized Fe_3_O_4_ NPs, which can undergo deprotonation in aqueous medium via the equilibrium shown in (Equation (3)).

−COOH + H_2_O ⇌ −COO^−^ + H_3_O^+^      pK_a_ ≈ 4.8 (3)

Finally, the presence of the alkyl chains of the oleic acid present in Fe_3_O_4_ NPs confers them a certain hydrophobic character, which would also contribute to their retention into the full nanosystem even though the polymer undergoes the hydrophilic-to-hydrophobic transition.

**Figure 4:**
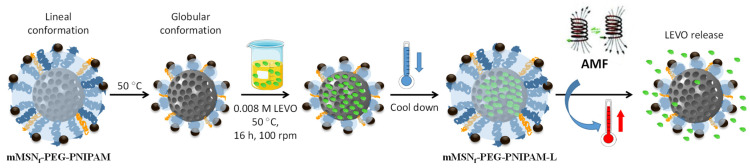
Schematic depiction of the experimental procedures carried out for the loading and release of LEVO from the mMSNf-PEG-PNIPAM nanosystem. Briefly, the nanosystem was soaked into a LEVO solution in EtOH at 50 °C to bring the polymer into globular conformation and allow LEVO loading into the mesopores. Then, the cool down favors the polymer adopting the expanded conformation to close the pores and prevent antibiotic release. Finally, the application of an AMF produces an increase in the local temperature that provokes a conformational change in the polymer from linear to globular, which triggers pore uncapping and LEVO release.

To investigate the stimulus-responsive behavior of this material and its bactericidal effect, the mMSN_f_-PEG-PNIPAM sample was loaded with a high-spectrum antibiotic such as levofloxacin (LEVO), generally used in the treatment of Gram-negative *E. coli* bacteria. The temperature chosen for the loading process was 50 °C to ensure the polymer to be in the globular conformation, allowing the antibiotic to enter the pores of the mesoporous silica. The loading process and stimuli-responsive release from the mMSN_f-_PEG-PNIPAM-L nanosystem is depicted in Figure 4.

### 3.2. In Vial Triggered Levofloxacin Release Assays

#### 3.2.1. Temperature as Release Trigger

**Figure 5:**
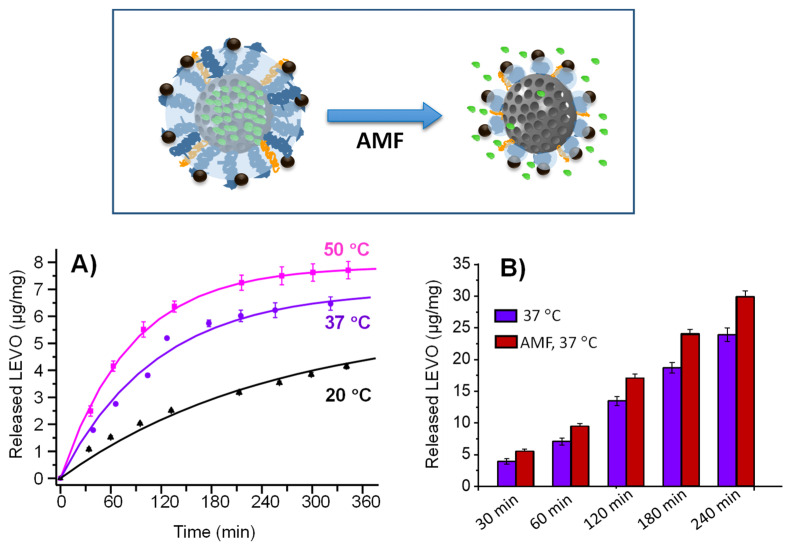
(**A**) LEVO release profile at different temperatures: 20, 37 and 50 °C. (**B**) Total LEVO release at different times at 37 °C (blue bars) and same conditions plus magnetic field exposition (red bars). Data are mean ± SD of the three replicas. The different conditions used during both experiments are detailed in Section 2.3.2.

To evaluate the release behavior of the mMSN_f_-PEG-PNIPAM-L nanosystem under physiological conditions (PBS 1×, pH 7.4), antibiotic release experiments were carried out in vial at different temperatures (20, 37 and 50 °C). Figure 5A shows the release profiles of LEVO for the different temperatures up to 6 h. All release profiles are characteristic of the typical diffusion model of MSN materials. Thus, the release profiles were fitted to the first-origin kinetics according to (Equation (4)) [98]:

*Y* = *A* (1−*e*^−*k t*^) (4)

where “*Y*” is the amount of LEVO released (µg LEVO/mg material) at time “*t*”, “*A*” the maximum amount of LEVO released (µg LEVO/mg material) and “*k*” the release rate constant. The parameters resulting from kinetics fitting are shown in Table S1.

The amounts of antibiotic released in the first hours were higher at 50 °C than at 20 and 37 °C (Figure 5A). Especially at 20 °C, the release was greatly hampered. At a time of 80 min, the antibiotic dose released at 50 °C was 1.4 times higher than that released at 37 °C and 3.1 times higher than that at 20 °C. This fact indicates that the thermosensitive polymer coating maintains its linear polymeric behavior at temperatures of 20 and 37 °C, being able to retain its charge at physiological temperature and releases it once the LCST value is reached. These differences are reflected in the release rate “*k*” values of 0.0118, 0.0087 and 0.0039 for 50, 37 and 20 °C, respectively.

Therefore, although a partial premature cargo release was not avoided at the physiological conditions in the absence of external stimuli (1.53 µg/mg material after 80 min of assay for 20 °C and 3.5 µg/mg material for 37 °C), the application of the AMF significantly increased the amount of antibiotic released to the delivery medium (4.8 µg/mg material after 80 min of assay). This fact would contribute to enhance the antimicrobial effect of the nanosystem, as was observed during the microbiological studies discussed in Section 3.3.

#### 3.2.2. Magnetic Field as Release Trigger

LEVO release experiments were also carried out by placing the mMSN_f_-PEG-PNIPAM-L nanosystem (1.96 mg/mL) in the hyperthermia device at 37 °C and applying an AMF (202 kHz and 30 mT). As a control, the same experiment in the absence of an AMF was also conducted. (Figure 5B). Frequencies that cause reasonable heating in hyperthermia are limited within the range 50 kHz < f < 1 MHz. Above 1 MHz, negative physiological damage may occur. On the other hand, the field amplitude is restricted to less than H_0_ < 15 kA/m [99]. Although the amplitude used in our case is higher, the f × H_0_ results (6 × 10^7^ Oe Hz) fall within the fitness range (<6.25 × 10^7^ Oe Hz) used in applications in small regions of the body of patients [70]. Therefore, the frequency and magnetic field conditions employed fall within the estimated safety range.

The sample subjected to an AMF presented a significantly higher released antibiotic dose than the sample without exposure in the same period of time (Figure 5B). At a time of 30 min, the percentage of levofloxacin released under magnetic field application to the study nanosystem was 43% higher compared with the nanosystem without magnetic field application. This different drug release behavior was also observed in different magnetic-field responsive MSNs-based drug delivery nanosystems incorporating SPIONs as hot spots [64], and may be due to the following factors: (i) the polymer transition temperature is reached faster in the particle environment when the field is applied; (ii) the vibrations of SPIONs when subjected to magnetic field can induce a higher release compared with incubator heating; and (iii) the localized temperature in the vicinity of the particle is higher than the global temperature [58,100], leading to greater diffusion of the antibiotic once the pore is opened. These mechanisms could act in concert, resulting in a higher release of LEVO in the presence of the AMF.

On the other hand, the values are very similar when comparing the amount of LEVO released (9.44 µg/mg material) at a time of 60 min under magnetic field application (Figure 5B) with the amount of total antibiotic released (7.87 µg/mg material) at 50 °C (Figure 5A), revealing that it is possible to cause the polymer transition under a magnetic field in a much shorter time. This fact would by supported by a recent study reported by Ovejero et al., who developed an innovative strategy, based on the linkage of fluorescent proteins as local thermal probes, to determine the specific local temperature in the surface of different sets of SPIONs using different AMF settings [61]. In the case of SPIONs obtained by thermal decomposition method exposed to a low-frequency AMF (100 kHz to 50 mT), a small increment in local temperature was produced. However, when SPIONs were submitted to more favorable AMF conditions (388 kHz and 15 mT), higher local temperatures up to ca. 70 °C were reached. In agreement with such studies, the AMF conditions used in the present work (202 kHz and 30 mT) could produce local temperatures above 50 °C in the surface of Fe_3_O_4_ NPs incorporated in the full nanosystem, which prompt the change in the polymer conformation and trigger faster LEVO release.

### 3.3. Microbiological Assays

**Figure 6:**
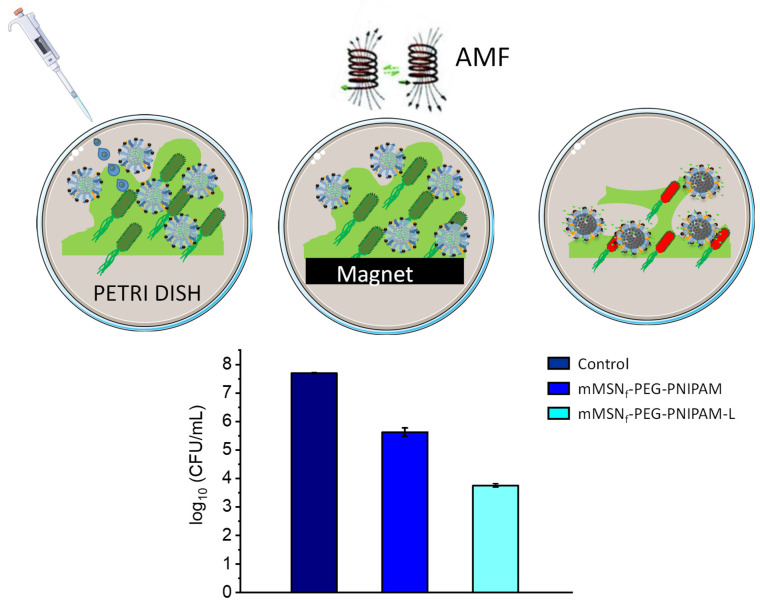
Bactericidal effect of mMSN_f_-PEG-PNIPAM-L nanosystem on *E. coli* biofilm. The graph shows log_10_ (CFUs per mL) after biofilm exposure to 200 µg/mL of the nanosystems for 16 h, compared with biofilm without nanoparticles as control (first bar). Data are mean ± SD of three independent experiments.

The bactericidal effect of the mMSN_f_-PEG-PNIPAM-L nanosystem on biofilms was studied to evaluate the combined effect of the antibiotic and the magnetic field on the same nanoplatform. For this purpose, pre-formed *E. coli* biofilms were exposed to a nanosystem concentration of 200 µg/mL. Prior to the application of the magnetic field, a neodymium magnet was placed underneath the Petri dish once the nanoparticles were added. This strategy aims at increasing the close contact between the magnetic nanocarrier and the biofilm surface and could even promote its penetration since, as previously reported, magnetic nanosystems can be guided to favor biofilm penetration in targeted antimicrobial therapies [8,101]. Once the magnetic field is applied, the antibiotic-free nanosystem (mMSN_f_-PEG-PNIPAM) produced a reduction in biofilm of two units in the log_10_ CFU/mL with respect to the bacterial control (Figure 6). This positive antibiofilm effect agrees with previously published reports on the ability of pure iron oxide NPs to generate heat by means of an AMF as a powerful tool in terms of inactivating antimicrobial cells [56]. With respect to the nanosystem loaded with the antibiotic mMSN_f_-PEG-PNIPAM-L and under the application of the alternating magnetic field, a large reduction in biofilm is observed, which is translated into four units of log_10_ CFU/mL (Figure 6).

Therefore, we have investigated the antibacterial efficacy of a new multicomponent nanosystem, noticing the enhanced effect produced by the combination of hyperthermia with the administration of antibiotics. The results obtained show relevant data in terms of bactericidal efficacy, being reflected in a viability reduction of almost four units in the log_10_ CFU/mL with respect to the control. It is worth noting that this remarkable antibiofilm effect is obtained at relatively small doses, envisaging this system as a promising nanoantibiotic. In addition, the versatility of this nanosystem relies on the possibility to load and release antibiotic cargoes of different families, which would allow the design of custom-made therapies depending on the characteristics of the infection to be treated. In this sense, this proof of concept allows us to bet on the performance obtained with this multicomponent nanosystem.

## 4. Conclusions

In this work, we report a new smart multicomponent nanodevice that combines the AMF-triggered delivery of a broad-spectrum antibiotic with magnetic hyperthermia for the local treatment of biofilm-associated bacterial infections as a proof of concept. This multicomponent nanosystem consists of mesoporous silica nanoparticles (MSNs), loaded with the broad-spectrum antibiotic levofloxacin, coated with a thermosensitive poly-*N*-isopropylacrylamide (PNIPAM)-based polymer and externally decorated with superparamagnetic iron oxide nanoparticles (SPIONs). The enhanced effect produced by the combination of magnetic hyperthermia and the controlled administration of the antibiotic leads to bacterial death and a significant bacterial biofilm reduction. The obtained results open future possibilities, and current studies are focused on increasing the dose of the nanosystem to improve the bactericidal efficacy, testing the nanodevice on other bacterial species and loading other molecules inside the pores to demonstrate the versatility of the nanosystem. Hence, a novel infection treatment is envisioned through an advanced nanosystem that can be locally administrated at the infection site, disrupting and eradicating the biofilm in the presence of an AMF. It is anticipated that this new biofilm eradication strategy could lead to a decrease in antibiotic use and thus a decrease in antibiotic resistance in the clinical setting.

## Figures and Tables

**Table 1:** Characteristics of the different MSNs synthesized in this work obtained by DLS and ζ-potential (data are mean ± SD of the five measurements).

Sample	D_H_ (nm)	ζ-Potential (mV)
MSN	160 ± 30	22 ± 4
MSN_f_	214 ± 13	43 ± 1
MSN_f_-PEG	221 ± 23	34 ± 2
MSN_f_-PEG-PNIPAM	255 ± 18	9 ± 1
mMSN_f_-PEG-PNIPAM	255 ± 20	−6 ± 1

## Data Availability

The data presented in this study are available on request from the corresponding authors.

## Supplementary Materials

The following are available online at https://www.mdpi.com/article/10.3390/pharmaceutics14010163/s1, Equipment; Figure S1: Scheme of synthesis of OA-Fe_3_O_4_ NPs and subsequent ligand exchange with citric acid to afford Fe_3_O_4_ NPs; Figure S2: NPs size distribution obtained by TEM images, XRD pattern and magnetization curve of OA-Fe_3_O_4_ NPs; Figure S3: EDS spectrum and atomic percentage of iron content in mesoporous silica nanosystem; Table S1: Kinetic parameters of LEVO release at different temperatures (20, 37 and 50 °C).
