# Supplementary material for: Superparamagnetic Iron Oxide Nanoparticles Decorated Mesoporous Silica Nanosystem for Combined Antibiofilm Therapy
Source: Pharmaceutics. 2022 Jan 11;14(1):163. doi: 10.3390/pharmaceutics14010163 (PMC8778149; doi:10.3390/pharmaceutics14010163)
Supplement: Supplementary file 1 [file pharmaceutics-14-00163-s001.zip › pharmaceutics-1530221-supplementary.pdf]

pharmaceutics

MDPI

Supplementary Materials: Superparamagnetic iron oxide nanoparticles decorated mesoporous silica nanosystem for combined antibiofilm therapy

Elena Álvarez, Manuel Estévez, Alvaro Gallo-Cordova, Blanca González, Rafael R. Castillo, María del Puerto Morales, Montserrat Colilla*, Isabel Izquierdo-Barba*, María Vallet-Regí*

Supplementary Materials Summary:

Equipment; Figure S1: scheme of synthesis of OA-Fe_3O_4 NPs and subsequent ligand exchange with citric acid to afford Fe_3O_4 NPs ; Figure S2: NPs size distribution obtained by TEM images, XRD pattern and magnetization curve of OA-Fe_3O_4 NPs; Figure S3: EDS spectrum and atomic percentage of iron content in mesoporous silica nanosystem; Table S1: kinetic parameters of LEVO release at different temperatures (20, 37 and 50 °C).

Equipment

Powder X-ray diffraction (XRD) measurements were performed in a Philips X'Pert diffractometer with Bragg-Brentano geometry operating with Cu Kα radiation (wavelength 1.5406 Å) at 40 kV and 20 mA (Philips Electronics NV, Eindhoven, Netherlands). XRD patterns were collected in the 2θ range between 10° and 90° with a step size of 0.04° and contact time of 5 s per step.

Electrophoretic mobility measurements for the materials were used to calculate the zeta-potential (ζ) values of the nanosystems. Measurements were performed in a Zetasizer Nano ZS (Malvern Instruments Ltd., Worcestershire, United Kingdom) equipped with a 633 nm "red" laser. For this purpose, 0.1 mg/mL of nanoparticles in water were dispersed using vortex and ultrasound to get homogeneous suspensions. Measurements were recorded by placing *ca.* 1 mL of the suspension in a DTS1070 disposable folded capillary cells (Malvern Instruments). Dilutions of the initial suspension were performed if needed. The hydrodynamic size of the nanoparticles was measured by Dynamic Light Scattering (DLS) with the same Malvern instrument. Values presented are mean ± SD from triplicate measurements

Transmission Electron Microscopy (TEM) and Energy Dispersive X-Ray Spectroscopy (EDS) were carried out with a JEOL JEM 2100 instruments (JEOL Ltd., Tokyo, Japan) operated at 200 kV, respectively, and equipped with a CDD camera (KeenView Camera). Sample preparation was performed by dispersing *ca.* 1 mg of sample in 1 mL of ethanol followed by sonication in a low power sonicator bath (Selecta, Spain) for 5 min, and subsequent deposition of one drop of the suspension onto carbon-coated copper grids. Images of SPIONs were obtained in a transmission electron microscope JEOL JEM 1100 (JEOL Ltd., Tokyo, Japan) operated at 100 keV from their colloidal suspensions deposited onto carbon-coated copper grids.

Fourier transformed infrared (FTIR) spectra were collected in a Thermo Nicolet Nexus spectrometer (Waltham, MA, USA) equipped with a Goldengate attenuated total reflectance (ATR) device.

The amount of LEVO released was determined by fluorescence spectroscopy using a BiotekPowerwave XS spectrofluorimeter, version 1.00.14 of the Gen5 program, using λ_{ex} = 292 nm and λ_{em} = 494 nm.

A vibrating sample magnetometer MagLabVSM (Oxford Instrument, High Wycombe, UK) was used to measure the magnetic properties of the nanoparticles. For the measurement, 25 μL of the sample with known concentration were placed on a piece of cotton wool, allowed to dry and pressed into a sample holder. The hysteresis loop was

measured at room temperature up to 5 T, and the magnetic saturation of the nanoparticles was obtained by extrapolating to 1/H = 0 the high field part of the magnetization curve.

Hyperthermia measurements were performed with a Fives Celes generator, model Nº 12118 M01 (Lautenbach, France). This system is equipped with a water-cooled copper coil of 50 mm diameter with 6 turns, an insulation chamber that allows a well-controlled environment and a fiber optic temperature sensor OSENSA`s FTX. Experiments were performed with at least 500 µL (1 g/L) of the sample placed in a vial in the middle of the heating coil under frequency of 202 kHz and external field amplitud of 30 mT.

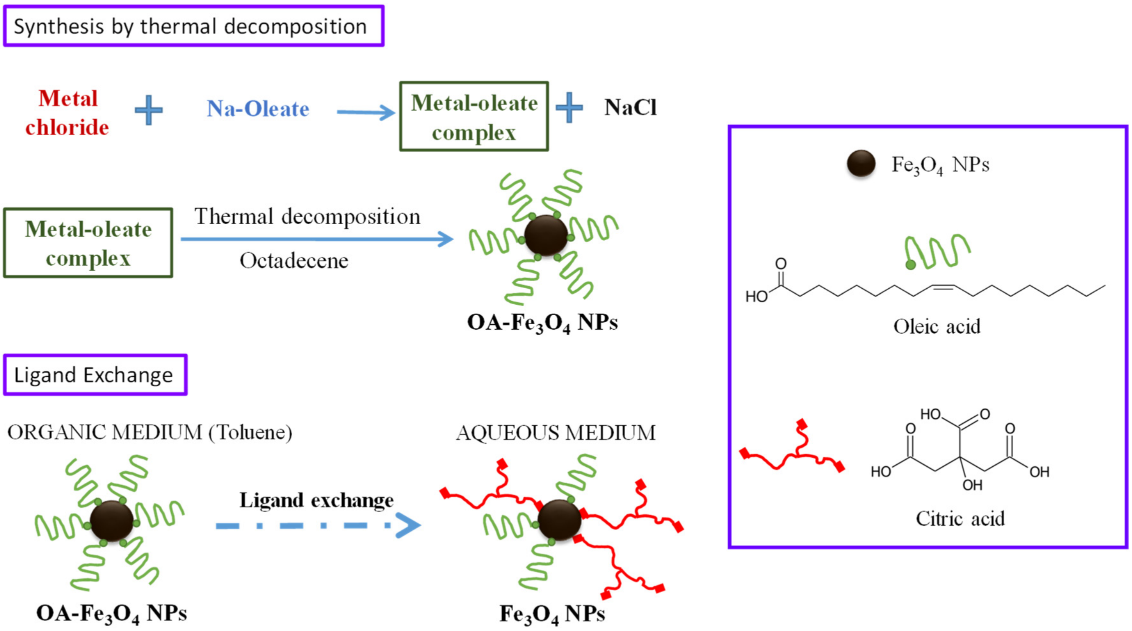

Figure S1. Scheme of synthesis of OA-Fe_3O_4 NPs and subsequent ligand exchange to afford Fe_3O_4 NPs colloidally stable in water media.

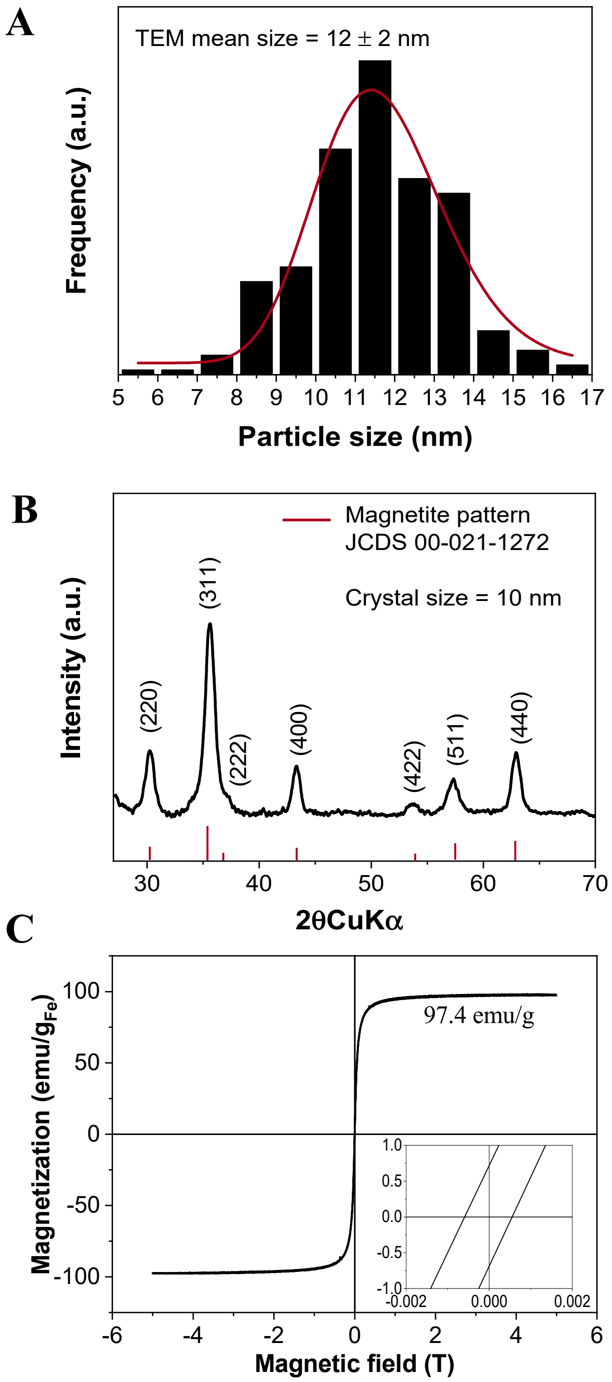

Figure S2. NPs size distribution obtained by statistical treatment of different TEM images (A), XRD pattern (B) and magnetization curve (C) of OA-Fe_3O_4 NPs (inset: magnification of the hysteresis loop).

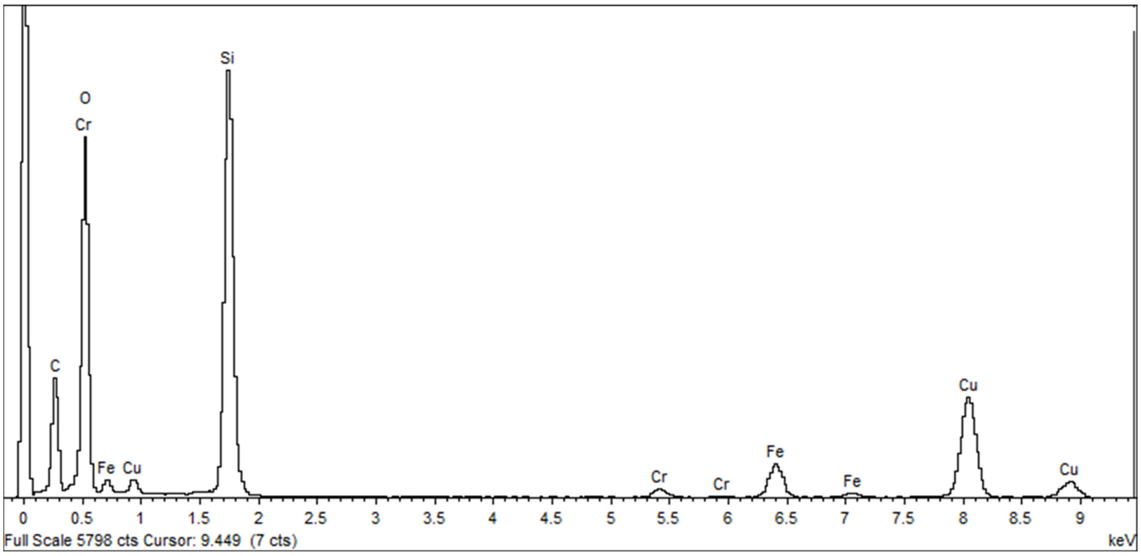

Figure S3. Energy-dispersive X-ray spectrum of mMNS$_f$-PEG-PNIPAM nanosystem. Atomic percentages of Fe and Si obtained are 7.2 ± 0.6 and 92.8 ± 0.4, respectively, resulting in a Fe/Si molar ratio of 0.0776. Data are mean ± SD of five measurements.

Table S1. Kinetic parameters of LEVO release from the **mMSN$_f$-PEG-PNIPAM-L** nanosystem at different temperatures using the $y = A(1–e^{-kt})$ equation.

Temperature (°C)	A (µg/mg) [a]	k (h^{-1}) [b]	R^2 [c]
20	5.7 ± 0.2	0.0039 ± 0.0003	0.98628
37	7.0 ± 0.2	0.0087 ± 0.0006	0.98829
50	7.84 ± 0.04	0.0118 ± 0.0003	0.99894

[a] A is maximum amount of LEVO released; [b] k is the release rate constant; [c] R is the regression coefficients of the different fits.
